# Maternal Dioxin Exposure Combined with a Diet High in Fat Increases Mammary Cancer Incidence in Mice

**DOI:** 10.1289/ehp.0901047

**Published:** 2009-12-09

**Authors:** Michele La Merrill, Rachel Harper, Linda S. Birnbaum, Robert D. Cardiff, David W. Threadgill

**Affiliations:** 1 Curriculum in Toxicology, Department of Genetics, Center for Environmental and Health Susceptibility, Lineberger Cancer Center, University of North Carolina–Chapel Hill, Chapel Hill, North Carolina, USA; 2 Experimental Toxicology Division, U.S. Environmental Protection Agency, Office of Research and Development/National Health and Environmental Effects Research Laboratory, Research Triangle Park, North Carolina, USA; 3 National Institute of Environmental Health Sciences, National Institutes of Health, Department of Health and Human Services, Research Triangle Park, North Carolina, USA; 4 Center for Comparative Medicine, Department of Pathology and Laboratory Medicine, University of California–Davis, Davis, California, USA; 5 Department of Genetics, North Carolina State University, Raleigh, North Carolina, USA

**Keywords:** COMT, CYP1B1, dioxin, high-fat diet, mammary cancer, puberty

## Abstract

**Background:**

Results from previous studies have suggested that breast cancer risk correlates with total lifetime exposure to estrogens and that early-life 2,3,7,8-tetrachlorodibenzo-*p*-dioxin (TCDD) exposure or diets high in fat can also increase cancer risk.

**Objectives:**

Because both TCDD and diet affect the estrogen pathway, we examined how TCDD and a high-fat diet (HFD) interact to alter breast cancer susceptibility.

**Methods:**

We exposed pregnant female FVB/NJ mice (12.5 days postcoitus) to 1 μg/kg TCDD or vehicle; at parturition, the dams were randomly assigned to a low-fat diet (LFD) or a high-fat diet (HFD). Female offspring were maintained on the same diets after weaning and were exposed to 7,12-dimethylbenz[*a*]anthracene on postnatal days (PNDs) 35, 49, and 63 to initiate mammary tumors. A second cohort of females was treated identically until PND35 or PND49, when mammary gland morphology was examined, or PND50, when mammary gland mRNA was analyzed.

**Results:**

We found that maternal TCDD exposure doubled mammary tumor incidence only in mice fed the HFD. Among HFD-fed mice, maternal TCDD exposure caused rapid mammary development with increased *Cyp1b1* (cytochrome P450 1B1) expression and decreased *Comt* (catechol-*O*-methyltransferase) expression in mammary tissue. Maternal TCDD exposure also increased mammary tumor *Cyp1b1* expression.

**Conclusions:**

Our data suggest that the HFD increases sensitivity to maternal TCDD exposure, resulting in increased breast cancer incidence, by changing metabolism capability. These results provide a mechanism to explain epidemiological data linking early-life TCDD exposure and diets high in fat to increased risk for breast cancer in humans.

Total lifetime exposure to estrogen (E2) is the single greatest environmental risk factor for breast cancer (Dunn et al. 2005). The classic pathway of E2-mediated carcinogenesis is through the estrogen receptor (ER), where E2 alters gene expression to increase cell proliferation (Currier et al. 2005). Consequently, it has been hypothesized that E2 metabolism acts to decrease breast cancer risk (Holcomb and Safe 1994). Yet, some E2 metabolites may increase breast cancer risk through DNA damage (Cavalieri and Rogan 2004; Cavalieri et al. 1997). E2 is metabolized by cytochrome P450 (CYP) 1B1 into reactive catechols that undergo redox cycling, resulting in oxidative stress, DNA adduct formation, and DNA mutations (Cavalieri and Rogan 2004; Chakravarti et al. 2001; Mitrunen and Hirvonen 2003). The phase II enzyme catechol-*O*-methyltransferase (COMT) mitigates this genotoxicity by inactivating the E2 catechols via *O-*methylation. 2,3,7,8-Tetrachlorodibenzo-*p*-dioxin (TCDD) and other aryl hydrocarbon receptor (AhR) agonists can modulate E2 activity through induction of E2-metabolizing CYP1A1 and CYP1B1 enzymes (Mitrunen and Hirvonen 2003). Thus, TCDD has the potential to modify breast cancer risk through alteration of ER-mediated proliferation or CYP1-mediated DNA damage (Mitrunen and Hirvonen 2003). The influence of TCDD exposure during early-life periods may be more pronounced because early-life estrogenic exposures appear to contribute to a greater risk of breast cancer than do exposures later in life (Dolinoy et al. 2007; Hilakivi-Clarke et al. 1999, 2000).

An industrial accident in Seveso, Italy, supports the link between early-life TCDD exposure and cancer risk. TCDD exposure was positively associated with breast cancer risk only in women who were infants up to 40 years of age at the time of the accident (Warner et al. 2002). Early-life TCDD exposure, particularly perinatally, has also been associated with increased mammary tumorigenesis in several rodent models (Brown et al. 1998; Desaulniers et al. 2001). Paradoxically, although greater E2 exposure in adolescents with early menses contributes to increased breast cancer risk (Vihko and Apter 1984), delayed pubertal breast differentiation may also increase breast cancer risk. Rodent models have shown that perinatal TCDD exposure increases mammary tumor risk through altered mammary differentiation (Brown et al. 1998; Fenton et al. 2002), which extends the period in which the rapidly proliferating progenitor cells of the terminal end buds (TEBs) are susceptible to carcinogenic insult (Birnbaum and Fenton 2003). A similar developmental delay has been reported in humans; as serum TCDD concentrations increase in either prenatal or premenarcheal samples, the timing of pubertal breast development is delayed (Den Hond et al. 2002; Leijs et al. 2008).

Like TCDD and E2 exposures, obesity may have age-specific or developmental-stage–specific effects on breast cancer risk (De Assis and Hilakivi-Clarke 2006). Obesity may also modify breast cancer risk through increased persistence of lipophilic TCDD in adipose tissue, including mammary stroma (Emond et al. 2006). Consequently, mammary glands of obese individuals are likely exposed to greater TCDD levels than in their lean counterparts (Emond et al. 2006; Harrad et al. 2003; Hooper et al. 1998; Michalek and Tripathi 1999). Because obese individuals retain more TCDD, maternal TCDD exposure may result in altered susceptibility to breast cancer among their offspring. In the present study we used the 7,12-dimethylbenz[*a*]anthracene (DMBA) mouse model of breast cancer to examine the mechanistic basis of how maternal TCDD exposure and obesity-associated high-fat diets (HFDs) increase cancer risk. We found that the combined effect of maternal TCDD exposure and an HFD increases mammary cancer risk through alterations in metabolism capability and the rate of breast development.

## Materials and Methods

### Mice and experimental design

We used a randomized 2 × 2 factorial design with the TCDD-responsive mouse strain FVB/NJ (Jackson Laboratory, Bar Harbor, ME). At 12.5 days postcoitus, nulliparous dams were administered 1 μg/kg TCDD orally (*n* = 13 dams; 1.8–1.9 μL TCDD solution/g mouse; 99.9% purity; Ultra Scientific, North Kingstown, RI) or an equivalent volume of vehicle (*n* = 14 dams; 95%/5% olive oil/toluene by volume; 99.9% purity; Sigma-Aldrich, St. Louis, MO). Litters were assigned to the HFD (*n* = 13 litters; D12451; Research Diets, New Brunswick, NJ) or control low-fat diet (LFD; *n* = 14 litters; D12450B; Research Diets) from postnatal day (PND) 0 (parturition) until euthanasia. The HFD was 4.73 kcal/g (20% protein, 35% carbohydrate, and 45% fat by total kcal), and the LFD was 3.85 kcal/g (20% protein, 70% carbohydrate, and 10% fat by total kcal). The primary differences between the matched diets are decreased cornstarch and sucrose and increased maltodextrin and lard in the HFD (291, 691, 400, and 1,598 kcal, respectively) compared with the LFD (1,260, 1,400, 140, and 180 kcal, respectively). Mice raised on the HFD were significantly heavier than those raised on the LFD beginning at preweaning (La Merrill et al. 2009a). Further, body weight, percent body fat, and fasting blood glucose of mice fed the HFD significantly increased with age relative to mice fed the LFD. However maternal TCDD exposure did not alter body weight, percent body fat, or fasting blood glucose (La Merrill et al. 2009a).

On PND4, all litters were culled to four pups, maximizing the number of female pups per litter. On PND21, all dams and any male offspring were removed from the cages. On PNDs 35, 49, and 63, all the female mice were administered 60 mg/kg DMBA orally (2.4 μL DMBA solution/g mouse; 95%/5% olive oil/toluene by volume; 98% purity; Sigma-Aldrich), hereafter referred to as the mammary cancer cohort. DMBA-treated mice were palpated for mammary tumors biweekly beginning on PND83.

In the parallel mammary gland cohort, mice were treated identically through PND49, inclusive of DMBA dosing, to examine potentially differential mammary gland morphology present when DMBA was administered in the mammary cancer cohort (PND35, PND49). mRNA expression was evaluated when mammary gland morphology was equivocal across exposure groups (PND50).

All mice were given water *ad libitum* in sterile ventilated cages in a facility approved by the American Association for the Accreditation of Laboratory Animal Care. Euthanasia was performed by CO_2_ asphyxiation on PNDs 35, 49, or 50, or when tumors were ≥ 1 cm in diameter or mice reached 11 months of age, whichever came first. All mice were treated humanely and with regard for alleviation of suffering, and all studies were approved by the University of North Carolina–Chapel Hill Institutional Animal Care and Use Committee.

### Histological analyses

Mammary tumors from the mammary cancer cohort mice were bisected at necropsy. One-half was flash-frozen, and the other half was fixed overnight at 4°C in 4% paraformaldehyde before dehydrating, embedding in paraffin, and sectioning. We evaluated tissue sections (4 μm; stained with hematoxylin and eosin) for pathology.

To determine tumor expression of ERBB2 (v-erb-b2 erythroblastic leukemia viral oncogene homolog 2) in mammary cancer cohort mice, paraffin sections were placed onto Superfrost/Plus slides (Fisher Scientific, Pittsburgh, PA), and then deparaffinized and cleared. After inhibition of endogenous peroxidase activity in a solution of 3% hydrogen peroxide in methanol, sections were hydrated in graded alcohols to distilled water. Antigen retrieval was performed using high-temperature/high-pressure incubation in 0.01 mol/L citric acid buffer (pH 6.0) for 12 min. Slides were allowed to cool for 30 min in citric acid buffer and then transferred to phosphate-buffered saline (PBS) at pH 7.4. Universal blocking reagent (BioGenex, San Ramon, CA) was applied to sections and incubated for 30 min in a humidified chamber at room temperature. ERBB2 antibody (Neomarkers, Fremont, CA) and rabbit secondary antibody (Santa Cruz Biotechnology, Santa Cruz, CA) diluted in PBS without ovalbumin were incubated for 1 hr each, and then sections were rinsed in PBS.

On PND35 and PND49, inguinal mammary glands from mammary gland cohort mice were weighed, fixed, and stained with carmine alum to evaluate fat pad length, number of TEBs, and branch elongation according to published methods (Fenton et al. 2002).

### Gene expression

For RNA extraction, we flash-froze and pulverized the other half of each mammary tumor and a grossly normal gland—defined as a gland from which no palpable or visible (5× dissecting scope) foci were observed—from the same mouse in the mammary cancer cohort study. PND50 mammary glands from mice in the mammary gland cohort study were flash frozen, pulverized, and pooled within litter for RNA extraction (TRIzol Reagent, Invitrogen). We used the High-Capacity cDNA Archive Kit (Applied Biosystems Inc., Foster City, CA) to generate cDNA for polymerase chain reaction (PCR) analysis.

Real-time PCR was performed on 100 μg total RNA to assess relative transcript levels of *Cyp1a1*, *Cyp1b1*, *Insig1* (insulin induced gene 1), *Ccdn1* (cyclin D1), *Myc* (myelocytomatosis oncogene), *Egf* (epidermal growth factor), *Ereg* (epiregulin), *Esr1* (estrogen receptor 1), and *Esr2* (estrogen receptor 2) using Assays-on-Demand (Applied Biosystems) with *Gusb* (glucuronidase, beta) as the endogenous control and *K18* (keratin 18) as an epithelial-specific marker in PND50 mammary glands (Livak and Schmittgen 2001). Real-time PCR was also performed on *Cyp1b1* and *Comt* to determine relative transcript levels using *Tbp* (TATA box binding protein) as the endogenous control in mammary tumors and matched controls (Applied Biosystems). Mammary gland RNA collected from mammary gland cohort mice at PND50 and Mouse Universal Reference RNA (Stratagene, La Jolla, CA), was prepared for global gene expression analysis using the Low RNA Input Linear Amplification Kit (Agilent Technologies, Santa Clara CA). The resulting cRNA was labeled with either Cy3-CTP (Mouse Universal Reference RNA) or Cy5-CTP (PerkinElmer, Waltham, MA) and purified (RNeasy; Qiagen, Valencia, CA). We evaluated global gene expression using a 4 × 44,000 microarray (Agilent Technologies) (Syed and Threadgill 2006).

### Statistics

We evaluated lesion latency [defined as time (PND) to reach palpable lesion] and lesion aggression [defined as the time from first palpable mass to time of mass ≥ 1 cm in diameter (PND)] by the hazard ratio (HR), using a mixed survival model in SUDAAN 9.0 software (Research Triangle Institute, Research Triangle Park, NC), with litter as a random effect. Lesion incidence (defined as the proportion of mice with mammary lesions per litter) was analyzed with Fisher’s exact test (SAS, version 9.1.3; SAS Institute Inc., Cary, NC). Significance of immunohistochemistry florescence was determined using the method of Rosner et al. (2002). We used analysis of variance to evaluate the fixed effects of HFD and maternal TCDD exposure and their interaction on branch elongation, fat pad length, number of TEBs in PND35 and PND49 mammary glands, and *Comt*, *Ccdn1*, *Cyp1a1*, *Cyp1a2*, *Cyp1b1*, *Insig1*, *Myc*, *Ereg*, *Ahr*, *Egfr* (epidermal growth factor receptor), *Esr1*, and *Esr2* levels in PND50 mammary glands (Proc GLM; SAS). The fixed effects of tissue status (with tumor or normal) and maternal TCDD, their interaction, and litter as a random effect were modeled for *Comt* and *Cyp1b1* levels in mammary tumors and matched normal tissue (Proc GLM).

Microarrays were scanned on an Agilent scanner and analyzed using default settings of Feature Extraction version 9.1 (Agilent). We uploaded microarray raw data into the UNC Microarray Database (http://genome.unc.edu) and performed Log2 R/G Lowess normalization on the Cy3 and Cy5 channels. The lowess-normalized data was quantile normalized (Barbacioru et al. 2006; Yang and Thorne 2003). Differentially expressed genes were identified using false discovery rate (FDR) < 0.05 threshold (SAM software, Stanford University, Stanford, CA) as described by Tusher et al. (2001). Significant genes and their fold change values generated in SAM were imported into Ingenuity Pathways Analysis (IPA 6.5–1602; Ingenuity Systems Inc., Redwood City, CA), where they were mapped to corresponding gene objects in the Ingenuity Pathways Knowledge Base (IPKB). The curated IPKB generated functional analyses of differentially expressed genes using Fisher’s exact test to calculate the probability (*p*-value) that each biological function and/or disease assigned to the gene set was due to chance alone.

## Results

### Increased incidence of mammary tumors

**Figure 1:**
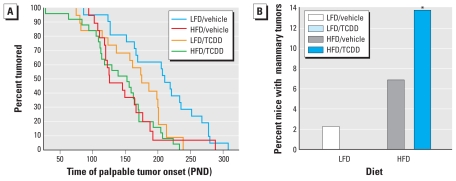
Effect of maternal TCDD and diet on palpable tumor latency and mammary tumor incidence in mammary cancer cohort mice treated with DMBA on PNDs 35, 49, and 63. (*A*) Time (PND) until first palpable tumor was detected (*n* = 28 litters). Tumor latency of HFD-fed mice treated with vehicle did not differ from that of HFD-fed mice treated with TCDD. Mean ± SD life spans were 255.1± 76.8 for LFD plus vehicle; 213.4 ± 83.8 for LFD plus TCDD; 167.2 ± 48.1 for HFD plus vehicle; and 178.8 ± 64.0 for HFD plusTCDD. (*B*) Percentage of mice with mammary tumors (*n* = 28 litters). *p < 0.0001 compared with HFD-fed mice treated with vehicle.

We found palpable lesions of both dermal and mammary origin after oral DMBA exposure, with 83% of mammary cancer cohort mice developing dermal lesions. Consistent with previous rat studies (Brown et al. 1998; Desaulniers et al. 2001), mammary cancer cohort mice fed LFD after maternal TCDD exposure had an average 59.7 days shorter latency to the first palpable lesions of either origin compared with those treated with vehicle (HR = 2.01081, *p* < 0.05; Figure 1A), although no mammary tumors arose in mammary cancer cohort LFD-fed mice exposed to TCDD (Figure 1B). The latency of the first palpable lesion of either origin was on average 80.5 days shorter for mammary cancer cohort mice fed HFD than for those fed LFD (HR = 0.0075, *p* < 0.01; Figure 1A), and these lesions grew faster (HR = 0.24827, *p* < 0.05; data not shown).

**Figure 2:**
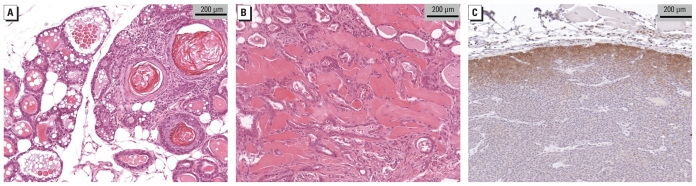
Effect of maternal TCDD and diet on mammary tumor pathology. (*A*) Adenosquamous carcinoma typical of the DMBA model. (*B*) Adenomyoepithelioma in DMBA-treated mice fed HFD and exposed to TCDD. (*C*) ERBB2-positive tumor in DMBA-treated mice fed HFD.

Because of the increased incidence of mammary tumors with either HFD or perinatal TCDD exposure seen in previous studies (Brown et al. 1998; Desaulniers et al. 2001; Hilakivi-Clarke et al 1997), we anticipated that HFD-fed mammary cancer cohort mice might have heightened susceptibility to maternal TCDD exposure. Consistent with this expectation, we observed a significant increase in mammary lesions of mice fed HFD after maternal TCDD exposure (*p* < 0.0001; Figure 1B). Mammary cancer cohort mice fed HFD had twice as many mammary lesions as mice fed LFD. Although no mammary lesions arose in litters exposed to TCDD and LFD, and one-third of unexposed litters fed HFD had mammary lesions, every litter exposed to both TCDD and HFD developed mammary lesions. Mammary lesions were primarily adenosquamous carcinomas, typical of the DMBA model (Figure 2A). However, several lesion types were clustered with respect to treatment. We found three adenomyoepitheliomas, a rare lesion in the DMBA model, exclusively in mammary cancer cohort mice perinatally exposed to TCDD and fed HFD (Figure 2B). In mammary cancer cohort mice treated with vehicle and fed HFD, we found two solid nodular ERBB2-positive tumors with zonation (Figure 2C) (Rosner et al. 2002).

### Transient alteration of pubertal mammary gland morphology

**Figure 3:**
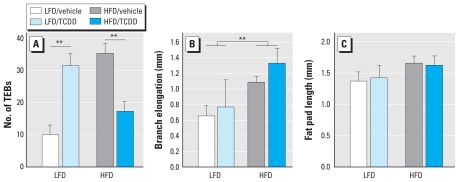
Interaction of maternal TCDD exposure and diet on mammary gland morphology at PND 35 in the mammary gland cohort. (*A*) Number of TEBs. (*B*) Branch elongation. (*C*) Fat pad length. Values shown are mean ± SE; animals are from a total of 20 litters. **p < 0.01.

Previous studies demonstrated a correlation among perinatal TCDD exposure, altered pubertal mammary differentiation, and increased mammary tumors in rats (Brown et al. 1998; Fenton et al. 2002). To determine whether alteration in mammary development could have contributed to differential cancer susceptibility in mice, we measured the effect of diet and TCDD exposure on mammary development in the mammary gland cohort. The combined effects of maternal TCDD and HFD exposures on the number of TEBs at PND35 deviated significantly from an additive model (*p* < 0.001 for interaction; Figure 3A); treatment with either TCDD or HFD increased the number of TEBs compared with mice treated with LFD and vehicle, whereas treatment with TCDD and HFD jointly decreased the number of TEBs. Among mammary gland cohort mice fed LFD, there was an average of 22 more TEBs per mouse with maternal TCDD exposure compared with vehicle. However, mammary gland cohort mice fed HFD had on average 18 fewer TEBs per mouse with maternal TCDD exposure (*p* < 0.01), a level comparable to that of the LFD group without TCDD treatment. We found no significant difference in the number of TEBs between HFD/vehicle mice compared with LFD/TCDD mice, or between HFD/TCDD mice compared with LFD/vehicle mice (Figure 3A).

Although the mean number of TEBs (Figure 3A), branch elongation (Figure 3B), and fat pad length (Figure 3C) were greater among mammary gland cohort mice fed HFD relative to LFD (26.3 vs. 18.6, 1.21 mm vs. 0.70 mm, and 1.64 mm vs. 1.39 mm, respectively), only branch elongation was significantly increased in the HFD group relative to the LFD group at PND35 (*p* < 0.05; Figure 3B).

Although perinatal TCDD exposure in rats prevents full differentiation of pubertal mammary glands (Fenton et al. 2002), we found the effects to be transient. By PND49, neither HFD nor maternal TCDD exposure affected the number of TEBs (data not shown). At PND49, fat pad length and ductal elongation were also unchanged by HFD and maternal TCDD exposure, and at PND50, we found no treatment-associated changes in expression of mammary morphology regulators *Egf* and *Ereg* (data not shown). These data indicate that mammary glands from mice exposed to both TCDD and HFD had a more rapid rate of maturation after PND35 than those from mice with only one treatment.

### Altered expression of genes involved with metabolism

Because mammary carcinogenesis may involve either E2- or ER-mediated proliferation or E2-metabolite–mediated genetic instability (Bolton and Thatcher 2008; Yager and Davidson 2006), we surveyed the transcriptome of mammary gland cohort mice for indications of these mechanisms. Analysis of the effect of diet on global gene expression demonstrated a subtle role for pubertal onset of obesity in modifying metabolism of carbohydrates, lipids, and proteins (*p* < 0.01), as reflected in the differential expression of 134 genes [FDR < 0.05; see Supplemental Material, Table 1 (doi:10.1289/ehp.0901047)]. Cancer was the disease/disorder category most significantly enriched (*n* = 20 differentially expressed transcripts; see Supplemental Material, Table 2) by mRNA due to HFD (*p* < 0.0001). However, most of these transcripts were down-regulated by diet, whereas only *Lrp1* (low density lipoprotein receptor-related protein 1) and *Aldh1a1* (aldehyde dehydrogenase family 1, subfamily A1) were modestly up-regulated by HFD, 1.6- and 1.8-fold, respectively (see Supplemental Material, Table 1). *Lrp1* was also the only gene among those associated with DNA damage that was significantly enriched by HFD (*p* < 0.01). Cellular proliferation (*p* < 0.01) was significantly enriched in the differential expression pattern caused by HFD. All genes associated with cell proliferation were down-regulated, suggesting that HFD may have reduced proliferation. No gene expression changes were significantly altered at the FDR < 0.05 level by maternal exposure to TCDD.

**Figure 4:**
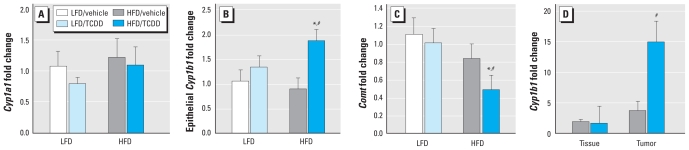
Effects of maternal TCDD and diet on E2 metabolism–associated gene expression. (*A*) Fold change in *Cyp1a1* expression in mammary glands of the mammary gland cohort (*n* = 20 litters, pooled within litter). (*B*) Fold change in epithelial *Cyp1b1* expression in mammary glands of the mammary gland cohort (*n* = 20 litters, pooled within litter). (*C*) Fold change in *Comt* expression in mammary glands of the mammary gland cohort (*n* = 20 litters, pooled within litter). (*D*) Fold change in tumor *Cyp1b1* expression relative to matched normal mammary gland tissue from HFD/vehicle of the mammary cancer cohort (*n* = 10 mammary tumors and 9 matched normal glands). *p < 0.05 compared with LFD/vehicle. ^#^*p* < 0.05 compared with HFD/vehicle.

We used quantitative real-time PCR to further examine the influence of TCDD and HFD on transcription associated with E2 metabolism and proliferation. We examined expression of *Cyp1a1*, *Cyp1b1*, and *Comt* as indicators of altered E2 metabolism capability in the mammary gland cohort mice. *Cyp1a1* expression was not altered by any treatment (Figure 4A). Exposure to HFD/TCDD increased epithelial *Cyp1b1* mRNA expression (Figure 4B) and decreased *Comt* mRNA expression (Figure 4C) in mammary glands relative to HFD/vehicle, LFD/vehicle, or LFD/TCDD mice (*p* < 0.05), but TCDD had no effect on expression in mice fed LFD. *Cyp1b1* was also increased in mammary tumors compared with matched normal mammary glands in the mammary cancer cohort (*p* < 0.05; Figure 4D). Among mammary tumors, *Cyp1b1* was elevated substantially by maternal TCDD exposure relative to vehicle (*p* < 0.05; Figure 4D). As opposed to E2 metabolism indicators, we observed no treatment-associated changes for the expression of the E2/ER proliferation indicators *Ccdn1*, *Myc*, *Egfr*, *Esr1*, or *Esr2* in mammary glands in the mammary gland cohort (data not shown). Together, these data support metabolism, and not E2/ER-mediated proliferation, as the likely mechanism underlying increased incidence of cancer caused by a combination of TCDD and HFD exposures.

## Discussion

Human and rat studies suggest that early-life TCDD exposure increases mammary cancer incidence (Brown et al. 1998; Desaulniers et al. 2001; Warner et al. 2002), which we confirmed in the present study in HFD-fed mice. There is substantial evidence that maternal estrogenic exposures, such as TCDD, HFD, and obesity, increase E2-responsive cancer incidence in adult offspring (Brown et al. 1998; Ho et al. 2006; Li et al. 1997; Prins et al. 2008). However, the mechanism by which this occurs is unknown. In the present study we investigated the mechanism by which HFD and maternal TCDD exposure increase breast cancer risk.

In the present study, DMBA-treated mice fed HFD had increased mammary tumor incidence and shortened latency relative to those fed LFD. We have shown previously that LFD has significantly greater estrogenic activity than does HFD, eliminating a direct role of diet on HFD-associated mammary tumor phenotypes (La Merrill et al. 2009a). HFD significantly down-regulated many of the genes found to be up-regulated in a lipid metabolic gene network associated with human breast cancer (e.g. *Acyl*, *Insig1*, *Elovl6*, *Fasn*, *Scd*) (Pitroda et al. 2009). Yet the two HFD up-regulated transcripts identified as significantly associated with cancer by the IPKB are also involved in lipid metabolism. *Aldh1a1*, which metabolizes products of lipid peroxidation (Alnouti and Klaassen 2008), has been found to be up-regulated in human breast cancer samples (Wilson et al. 2002). Similarly, *Lrp1* is involved with breast cancer cell migration (Dedieu et al. 2008), DNA damage (Ling et al. 2007), and obesity (Hofmann 2007). Thus, change in gene expression due to HFD is consistent with the genotoxicity hypothesis.

We have also shown that HFD increased adiposity and fasting blood glucose from puberty throughout life in these same mice (La Merrill et al. 2009a). Thus, the mammary cancer cohort mice fed HFD have greater adiposity and risk for metabolic syndrome than those fed LFD. Although increased adiposity slows TCDD elimination, extending its half-life (Michalek and Tripathi 1999), pharmacokinetic models designed to account for this predict that virtually no TCDD is stored in the mice at the time of tumor onset (Emond et al. 2006). Further, because *Cyp1a1*, highly inducible by TCDD, was not significantly induced by TCDD at PND50 in the mammary gland cohort, it is likely that very little TCDD remained in the mice at that age.

Obesity increases postmenopausal breast cancer in women (Lorincz and Sukumar 2006), which may occur because of heightened E2 production in mammary adipose tissue (Simpson 2003). Further, several studies have found that increased adiposity interacts with alleles of E2-metabolizing enzymes to increase breast cancer risk (Kocabas et al. 2002, 2005; Thompson et al. 1998). Thus the doubled mammary tumor incidence in HFD-fed mice exposed to maternal TCDD here may reflect higher steady-state levels of both E2 and its toxic metabolites.

**Figure 5:**
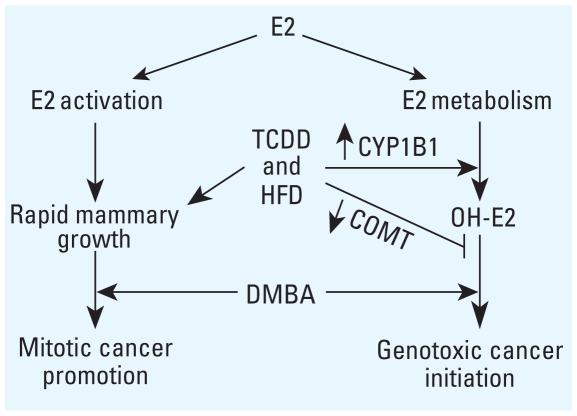
Model of E2-mediated breast cancer carcinogenesis. The combination of maternal TCDD and HFD increases *Cyp1b1* and decreases *Comt* expression, likely leading to enhanced genotoxic damage.

TCDD up-regulated epithelial *Cyp1b1* expression and decreased *Comt* expression in pubertal mammary glands from mammary gland cohort mice fed HFD, indicating a role of the E2 metabolism genotoxicity pathway in cancer etiology (Figure 5), consistent with studies showing a role for maternal *COMT* and *CYP1B1* in breast cancer risk of exposed daughters (Inoue et al. 1980; Sata et al. 2006). Although modest changes are common in constitutive *Cyp1b1*, environmental exposures infrequently change *Comt* expression; one rat model of aryl hydrocarbon exposure showed decreased hepatic *Comt* expression (Desaulniers et al. 2005). Human studies have suggested that high-activity *CYP1B1* alleles and low-activity *COMT* alleles are associated with increased risk of breast cancer (Wen et al. 2007) and other E2-responsive cancers (Nock et al. 2006; Sellers et al. 2005; Zimarina et al. 2004). Consequently, interactions between E2 exposures during key life stages as modeled here and the E2-regulating genes *Cyp1b1* and *Comt* may modify breast cancer risk (Mitrunen et al. 2001).

Maternal TCDD also substantially increased *Cyp1b1* expression in mammary tumors, suggesting that changes in mammary progenitor cells resulted in increased *Cyp1b1* that persisted through their expansion to tumors. However, TEB numbers were reduced in mice fed HFD and exposed to TCDD in the mammary gland cohort. Further, identically treated DBA/2J mice also have reduced TEB numbers at PND35 (La Merrill et al. 2009b). Because TEB number correlates with the carcinogenicity of DMBA (Russo and Russo 1978), we did not expect this TCDD delay of pubertal mammary growth. Our observations indicate that altered mammary morphology during cancer initiation (PND35) is not the primary mechanism for elevated tumor incidence caused by maternal TCDD and HFD exposure. However, mammary gland morphology was unchanged by diet and maternal TCDD at subsequent ages, suggesting rapid compensatory growth in mammary glands exposed to HFD and maternal TCDD. This rapid compensatory growth hypothesis is substantiated by the low *Comt* expression seen in these mice by Eriksson et al. (2005). Rapid compensatory mammary growth between the first and second dose of DMBA could have contributed to increased tumor incidence in two ways. Rapid growth might narrow the window of time available for repair of DNA damage potentially caused by altered E2 metabolism (Budzowska and Kanaar 2009; Chakravarti et al. 2001). Second, rapid mammary growth would facilitate expansion of *Cyp1b1*-expressing mammary epithelial cell populations into tumor cells. Although no human studies document the influence of dioxin on the tempo of puberty, the dioxin-induced delay in pubertal mammary growth and later mammary tumor incidence seen here has been demonstrated in human studies (Den Hond et al. 2002; Leijs et al. 2008; Warner et al. 2002).

## Conclusion

Maintenance of mice on HFD increased the effects of maternal TCDD on mammary cancer risk in offspring through alterations in metabolism capability. E2-like exposures, such as an obesogenic diet, combined with maternal TCDD exposure may increase the risk of breast cancer in the subsequent generation. The inconsistent relationships between *CYP1B1*, *COMT*, and breast cancer risk seen across epidemiology studies may reflect divergent risk associated with variable environmental E2 exposures (Justenhoven et al. 2007; Le Marchand et al. 2005; McGrath et al. 2004). This may understate the susceptibility to TCDD among overweight, genetically susceptible adolescents. Our results highlight the importance of using experimental animal models to evaluate the unique susceptibilities of varying subpopulations to breast cancer risk and to ensure that risk assessments in humans do not fail to take susceptible subpopulations into account.
